# Supplementary material for: Electrical Characterization of Cellulose-Based Membranes towards Pathogen Detection in Water
Source: Biosensors (Basel). 2021 Feb 21;11(2):57. doi: 10.3390/bios11020057 (PMC7927109; doi:10.3390/bios11020057)
Supplement: Supplementary file 1 [file biosensors-11-00057-s001.pdf]

biosensors

MDPI

Electrical Characterization of Nitrocellulose Membranes towards Bacterial Detection in Water †

Grégoire Le Brun[1,*], Margo Hauwaert [1], Audrey Leprince [2], Karine Glinel [3], Jacques Mahillon [2] and Jean-Pierre Raskin [1]

1 Institute of Information and Communication Technologies, Electronics and Applied Mathematics, UCLouvain, 1348 Louvain-la-Neuve, Belgium; margo.hauwaert@student.uclouvain.be (M.H.); jean.pierre.raskin@uclouvain.be (J.-P.R.)
2 Laboratory of Food and Environmental Microbiology, Earth and Life Institute, UCLouvain, 1348 Louvain-la-Neuve, Belgium; audrey.leprin@uclouvain.be (A.L.); j.mahillon@uclouvain.be (J.M.)
3 Institute of Condensed Matter and Nanosciences (Bio and Soft Matter), UCLouvain, 1348 Louvain-La-Neuve, Belgium; k.glinel@uclouvain.be
* Correspondence: gregoire.lebrun@uclouvain.be

Table S1. Results from the fitting of impedance data (Figure 5) to the simple electrical equivalent model used for the parallel-plate setup. All fit were accurate ($R^2 < 0.2$). Mean (μ) ± standard deviation (s. d.) on twelve measurements from two independent experiments.

Saline solution c_{NaCl} [mol/L]	R_{NC} [Ω]	C_{NC} [pF]	C_{baking} [pF]
	Mean (μ) ± s. d. (σ)	Mean (μ) ± s. d. (σ)	
**10^{-5} M**	202 923 ± 4855	9.028 ± 0.071	100
**10^{-4} M**	191 393 ± 2426	8.336 ± 0.024	100
**5×10^{-4} M**	164 160 ± 7400	8.819 ± 0.123	100
**10^{-3} M**	105 279 ± 3813	10.32 ± 0.13	100
**5×10^{-3} M**	55 512 ± 322	11.85 ± 0.051	100
**10^{-2} M**	23 422 ± 2096	16.08 ± 0.397	100
**10^{-1} M**	2165 ± 76	19.75 ± 0.431	100

Table S2. Results from the fitting of impedance data (Figure 6) to the simple electrical equivalent model used for the parallel-plate setup. All fit were accurate ($R^2 < 0.2$). Mean (μ) ± standard deviation (s. d.) on twelve measurements from two independent experiments.

Biological solution	R_{NC} [Ω]	C_{NC} [pF]	C_{baking} [pF]
	Mean (μ) ± s. d. (σ)	Mean (μ) ± s. d. (σ)	
**PBS:1000**	156 313 ± 1580	8.223 ± 0.025	100
***B. thuringiensis* 10^8 CFU/mL**	118 317 ± 2426	9.312 ± 0.018	100
